# Repeated Training with Augmentative Vibrotactile Feedback Increases Object Manipulation Performance

**DOI:** 10.1371/journal.pone.0032743

**Published:** 2012-02-27

**Authors:** Cara E. Stepp, Qi An, Yoky Matsuoka

**Affiliations:** 1 Department of Speech, Language, and Hearing Sciences, Boston University, Boston, Massachusetts, United States of America; 2 Department of Precision Engineering, The University of Tokyo, Tokyo, Japan; 3 Department of Computer Science and Engineering, University of Washington, Seattle, Washington, United States of America; Katholieke Universiteit Leuven, Belgium

## Abstract

Most users of prosthetic hands must rely on visual feedback alone, which requires visual attention and cognitive resources. Providing haptic feedback of variables relevant to manipulation, such as contact force, may thus improve the usability of prosthetic hands for tasks of daily living. Vibrotactile stimulation was explored as a feedback modality in ten unimpaired participants across eight sessions in a two-week period. Participants used their right index finger to perform a virtual object manipulation task with both visual and augmentative vibrotactile feedback related to force. Through repeated training, participants were able to learn to use the vibrotactile feedback to significantly improve object manipulation. Removal of vibrotactile feedback in session 8 significantly reduced task performance. These results suggest that vibrotactile feedback paired with training may enhance the manipulation ability of prosthetic hand users without the need for more invasive strategies.

## Introduction

Prosthetic limb technology has reached an advanced state, with increased degrees of freedom and light and compact form factors in products such as the i-LIMB hand (Touch Bionics Inc.) and the DEKA Arm (“Luke Arm”, DEKA Research and Development Corporation), along with advanced strategies for control, as reviewed in [1]. However, the majority of commercial products do not include sensory feedback, which could improve patient motor abilities. In fact, users of prosthetic hands have identified the addition of haptic feedback and relief from visual attention to perform functions as top design priorities [2], [3].

Real-time recording of prosthetic fingertip forces is already possible [4], [5], [6], [7], [8], and many groups have successfully integrated these technologies into prosthetic hands, e.g., the cybernetic hand [6], [7]. However, it is not yet clear how to translate this force feedback to users to optimally integrate the information for sensorimotor control. A variety of modes of delivery have been suggested [9], [10], [11], [12], [13], [14], [15], [16], ranging in complexity and invasiveness. More invasive approaches under investigation include electrocutaneous [17], peripheral nerve [18], [19], and cortical stimulation [20]. Sensory substitution refers to transformation of sensation across or within sensory systems, and the non-invasive substitution of remote vibrotactile feedback for fingertip force is an attractive proposal [5], [21], [22], [23]. Application of augmentative vibrotactile feedback at a remote healthy body site would rely on human neuroplasticity to integrate the feedback at the remote site for use in sensorimotor control. However, if successful, the non-invasive nature of this approach would allow for immediate wide-scale implementation among users of prosthetic hands [9], [10]. Unfortunately, past research utilizing vibrotactile feedback has been at best inconclusive about its effect on motor performance [5], [21], [23], [24].

Some previous studies have shown positive effects of vibrotactile feedback [5], [23]. Mann and Reimers showed that an individual using the Boston Arm was able to improve the accuracy of arm positioning using vibrotactile stimulation on his residual limb to signal tactual display of limb angle [23]. Likewise, five users of myoelectric prosthetic hands were able to decrease contact forces during a simple object grasp task when vibrotactile feedback related to contact force was available. Conversely, other studies have not found vibrotactile stimulation to be effective feedback [21], [24]. During gripping trials in which unimpaired participants attempted to match force production from a previous grip using a robotic arm, the five participants provided with both visual feedback and vibrotactile feedback related to the force applied did not show decreased error relative to the five participants who received visual feedback alone [24]. Furthermore, eight unimpaired individuals using a myoelectric prosthesis simulator to complete an interactive force-matching task did not show a consistent reduction in error with the addition of vibrotactile feedback on the upper arm [21].

More recently, our group has designed a simple virtual interface in which visual and haptic feedback can be experimentally controlled in order to quantitatively examine and compare possible methods of delivery of sensory feedback. In a recent study, eighteen unimpaired individuals participated for 2.5–4 hours using this interface to manipulate a virtual object with visual and vibrotactile feedback at four body sites (finger, arm, neck, and foot), presented in a random order [25]. We found that the effects of learning over the course of the experiment overshadowed the effects of supplying feedback at different stimulation sites. In fact, performance showed a strong learning effect across time, with all participants showing large increases in ability throughout their participation. However, training effects appeared to saturate by the end of the single session.

Because this previous study was performed in a single session, it was unclear whether the apparent saturation in performance was a result of participants having reached their steady-state ability or the effect of fatigue and boredom from many continuous hours of experimentation. Previous studies have shown that motor performance is dependent both on the total training time as well as the total elapsed time, with additional improvements in performance seen in follow-up testing with no additional training [26], [27]. Thus, multi-day training with vibrotactile feedback could show increased benefit on motor performance.

No previous study has tested participants on their ability to incorporate visual and remote vibrotactile feedback for object manipulation past a single session of interaction, so the role of experience and training is currently unknown. Thus, discrepancies in performance noted in previous studies could be the result of training time. Vibrotactile stimulation is cheap, non-invasive, and could be easily implemented into existing prosthetic technologies as augmentative sensory feedback [9], [10]. Thus, if training can significantly alter the abilities of individuals to incorporate remote vibrotactile feedback into sensorimotor control, vibrotactile stimulation may represent a viable feedback modality for prosthetic hand users.

The focus of this paper is to determine the performance trajectory of individuals training to use visual and augmentative vibrotactile feedback to perform a virtual object manipulation task. We used a previously-designed robotic and virtual interface to study object manipulation in which both visual, direct haptic, and vibrotactile feedback could be experimentally controlled [25]. This virtual task allowed us to study the effects of vibrotactile feedback as a substitute for typical, direct force feedback in healthy participants. We compared virtual object manipulation across eight sessions within a two-week period. During the final (8^th^) session, participants were not given the vibrotactile feedback, but were asked to perform the task using vision alone in order to identify the relative performance contributions of increased skill at completing the task versus increased ability to perceive and use the vibrotactile feedback. Performance was also compared as a function of a simultaneous cognitive load given the requirement that sensory feedback schemes must be applicable to everyday life of prosthesis users. We hypothesized that participants would show increases in performance throughout training until reaching steady-state performance, and that removal of the vibrotactile feedback in the 8^th^ session would result in decreased performance. Although it is possible that motor skill learned using vibrotactile feedback would be maintained after removal of the feedback, we rather anticipated that participants would develop strategies to incorporate the vibrotactile cues to perform the task and that this reliance would cause a degradation from optimal performance when asked to perform the task with vision alone. We also hypothesized that overall task performance would be decreased during a simultaneous cognitive task as has been shown in our previous work in single-visit experiments [25].

## Methods

### Participants

Participants were ten right-handed adult volunteers (seven female, three male; mean age = 22.6 years, SD = 4.1 years). All participants reported normal hand function. Informed consent was obtained from all participants in compliance with the Institutional Review Board of the University of Washington and participants were compensated $10/hr for their time.

### Virtual Environment

**Figure 1:**
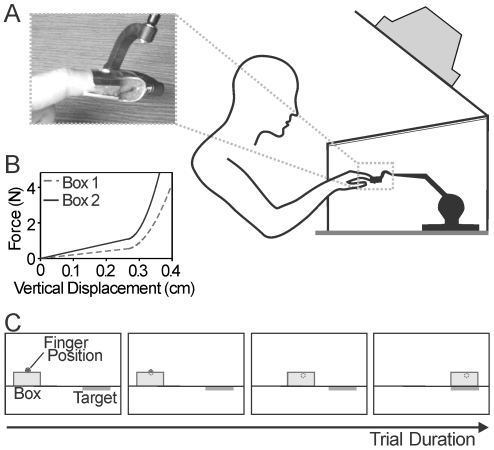
Experiment Methodology. Panel A shows the physical set-up of the experiment. Participants interacted with the virtual environment by placing their right index finger into a custom splint attached to the PHANTOM. Participants sat in front of the projection system and their hand was free to move about the 3D workspace. The PHANTOM was located inside a projection system, consisting of a frame above the PHANTOM, supporting an inverted video monitor. Panel B shows the force-displacement curves for the stiffnesses of box 1 and box 2. Panel C shows a schematic of the visual feedback supplied to the participant during a single successful trial. Participants attempted to move a box from the left of the screen to a target position by pushing down on the box and sliding it to the right. Finger position was indicated by a small sphere and was occluded during penetration of the box. Deformations of the box were not shown.

The experimental task was to apply appropriate normal force to a virtual object to allow for translation across a horizontal surface, and then to drag it to a target as quickly as possible without breaking it (see Fig. 1). This task was chosen in light of the known difficulties of prosthetic hand users with appropriately applying normal force to delicate objects, such as picking up and manipulating a disposable plastic cup [28]. The task was purposefully implemented to be difficult for participants to perform so that changes in ability would not be masked.

Participants interacted with the virtual environment by placing their right index finger into a custom splint attached to the end effector of a PHANTOM Premium 1.0 robotic device (Sensable Technologies, Inc., Woburn, MA), which was used to measure the position of the tip of the finger. The PHANTOM was located inside a projection system, consisting of a frame above the PHANTOM, supporting an inverted video monitor. The video monitor was positioned at 45° toward the participant, and a mirror was placed between the virtual environment and the monitor to permit reflection of images from the monitor to the user (see Fig. 1). Participants sat in front of the projection system with their right hand free to move about the 3D workspace.

The virtual environment was programmed in C++, with graphics driven by OpenGL. During interaction, one of two possible virtual objects appeared at the left end of the workspace (see Fig. 1). The upper surface of these objects (referred to as boxes) had distinct stiffness characteristics. These box stiffness functions were scaled versions of a fit to the force-displacement curve acquired empirically by pushing on a disposable plastic cup. Two objects were used in order to discourage participants from overtraining on one system. The difference between the two boxes was signaled to the participant by box color: box 1 was blue and box 2 was red. The stiffness characteristics of each box are shown in Figure 1 and were defined as:
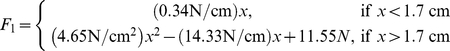


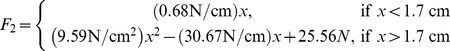
The virtual normal force of box 1, *F_1_*, and the virtual normal force of box 2, *F_2_*, were defined with *x* as the displacement (in cm) of the finger into the box in the normal direction (vertical). The stiffness of box 2 was greater than the stiffness of box 1.

*F_move_*, the minimum normal force required to overcome static friction between the box and the table, was arbitrarily defined as 1.2 times the virtual force at the displacement of 1.7 cm. The virtual force threshold to “break” each box, *F_break_*, was defined as 0.75N greater than *F_move_*. Virtual normal force applied to the box between *F_move_* and *F_break_* allowed the participant to slide the object to a target located 30 cm to the right of the workspace. The window of forces between being able to move the box and breaking the box was a constant 0.75N, regardless of whether it was box 1 or box 2. The difference in stiffness between the two boxes and this constant allowable force window resulted in a larger allowable displacement of the finger in the direction of the virtual normal force for box 1 than for box 2 (2.7 mm for box 1 and 1.6 mm for box 2).

Participants received visual feedback during each trial consisting of a real time depiction of the location of the finger in the virtual environment, and the current position of the box (see Fig. 1). Finger position was indicated by a small sphere (5 mm radius) that was gradually occluded during penetration of the box. Deformations of the box were not shown. This level of visual feedback was provided to the participants to approximate the real-life visual feedback of task performance available to users of prosthetic limbs during object manipulation. Because only the vertical and horizontal movements were relevant for the task, finger location in the third dimension of the virtual environment was displayed as a constant value and did not affect the motion of the box.

### Experimental Design

Individuals participated in eight sessions on eight separate days in a two-week period. Sessions 1–7 consisted of both visual and remote vibrotactile feedback. During session 8 participants received no vibrotactile feedback, but were asked to complete the task using vision alone. Each session consisted of 40 trials of interaction with the virtual system, so that each participant completed a total of 320 trials over the eight sessions.

The 40 trials in each session were pseudo-randomized as a function of box (1, 2) and cognitive task (on, off). Trials ended when the box reached the target or was broken. Participants were encouraged to take breaks between any trials to avoid fatigue, but rarely did. Each session generally took between 30–45 min including any breaks.

### Vibrotactile Feedback

Vibrotactile stimulation at 250 Hz was provided using a C2 tactor (Engineering Acoustics, Inc.) mounted to the right lateral upper arm and secured with an elasticized cloth bandage. A 250-Hz carrier frequency was used since human glabrous skin has been shown to be maximally sensitive to vibrotactile stimulation at this frequency [29], [30]. During interaction with the virtual environment, increases in virtual normal force were linearly translated to increases in the amplitude of continuous vibrotactile stimulation. The maximum amplitude of vibration used was approximately 400 µm, which corresponded to the force required to break box 2 (2.14 N).

### Cognitive Task

In order to determine the motor task performance during a simultaneous cognitive load, an auditory 2-back task was used [31]. During the test, participants listened to strings of 16 random digits and responded verbally to identify any numbers repeated with a single intervening number. Numbers were presented at 1 Hz. Prior to experimentation, participants practiced 20 sets of this task without simultaneously performing the motor task to ensure comprehension. During experimentation, participants were asked to complete the cognitive task while simultaneously completing the motor task. Because each number string was of a specific finite length and the length of each trial was variable, completion of the entire sixteen digits of the cognitive task was not achieved if the box was broken in less than 16 s.

Noise-canceling headphones (Bose, Framingham, MA) were worn by participants during experimentation to present the stimuli for the cognitive task, and to provide low-level masking noise. The masking noise and noise-canceling headphones were used to ensure that participants were not using any auditory feedback from the tactor to complete the motor task, since the vibrotactile feedback was provided in the range of human hearing.

### Analysis

Box displacement (total distance toward the target that the participant was able to translate the box during the trial) and average box velocity (box displacement normalized by trial duration) were used as performance variables and were determined for each trial using custom software in MATLAB (Mathworks, Natick MA). Statistical analysis was performed using Minitab Statistical Software (Minitab Inc., State College, PA). A three factor repeated measures analysis of variance (ANOVA) was performed to assess the effects of session, cognitive task, and box, as well as the interactions of cognitive task × session, box × session, and box × cognitive task on the two performance variables. Post hoc two-sided Tukey's Simultaneous tests were used when appropriate. All statistical analyses were performed using an alpha level of 0.05 for significance.

## Results

**Figure 2:**
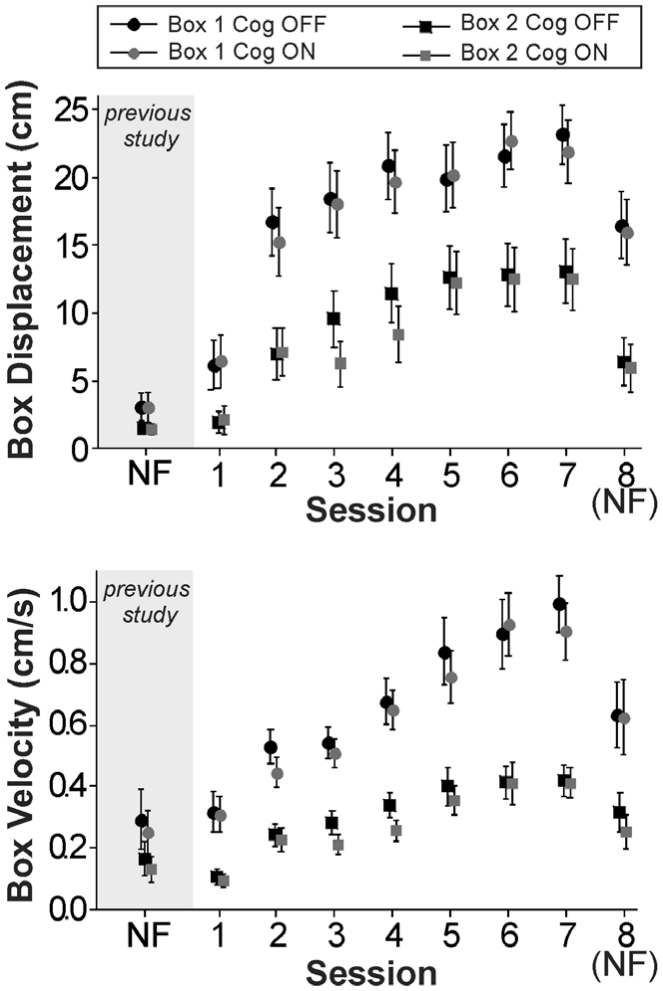
Effects of box, cognitive task, and session on box displacement and velocity. Participants were asked to move one of two possible boxes (box 1 or box 2) from the left of the screen to a target position by pushing down on the box and sliding it to the right. Half of trials were performed while participants were completing a simultaneous cognitive task (Cog ON) and during the rest participants completed the motor task alone (Cog OFF).Box displacement was defined as the total distance toward the target that the participant was able to translate the box during the trial and average box velocity was defined as the box displacement normalized by trial duration. Markers indicate data means and error bars mark 95% confidence bands of the mean. During sessions 1–7 participants were provided with vibrotactile feedback proportional to the normal force they were applying to the box as well as visual feedback. During session 8 they completed the task using visual feedback alone (NF = no feedback). The shaded area corresponds to data from a previous study [22] in which participants trained using visual feedback alone in a single session.

Out of 3200 combined trials in the eight sessions of participation, participants were able to successfully move the box to the target 817 times (25.5% of attempts). During successful trials, the average distance achieved was the full range of the task (30 cm) and the average velocity was 0.84 cm/s (SE = 0.015 cm/s). During unsuccessful trials, the average distance achieved was 7.54 cm (SE = 0.18 cm) and the average velocity was 0.35 cm/s (SE = 0.007 cm/s). Figure 2 shows the effects of feedback, block, and cognitive task on box displacement and velocity.

Results of the ANOVA on box displacement indicated statistically significant (*p*<0.05) effects of box, cognitive task, session, and the interaction box × session (see Table 1). Post hoc testing showed that participants could move box 1 significantly further than box 2 (t = −25.9, DF = 1599, *p_adj_*<0.001, *d* = −0.75) with a mean displacement of 17.7 cm (SE = 0.32 cm) versus a mean of 8.8 cm (SE = 0.27 cm). Presence of the simultaneous cognitive task significantly decreased mean displacement (t = −2.04, DF = 1599, *p_adj_* = 0.04, *d* = −0.06) from 13.6 cm (SE = 0.31 cm) to 12.9 cm (SE = 0.31). Pairwise comparisons between the 8 sessions of experimentation (see Table 2) indicated several significant differences showing a general increase in box displacement as a function of session until reaching steady state at session 5. Session 1 had significantly lower displacement relative to all other sessions. Session 7 showed significantly increased displacement relative to sessions 1–4. Session 8 showed lowered displacement relative to sessions 4, 5, 6, and 7, but increased displacement relative to session 1. Trials with both box 1 and box 2 showed increases in displacement as a function of session, but greater increases were seen for box 1 than for box 2.

**Table 1:** ANOVA for Box Displacement.

Factor	DF	η_p_^2^	F	*p*
Session	7	0.133	85.4	<0.001
Cognitive Task	1	<0.001	4.2	0.041
Box	1	0.151	672.1	<0.001
Cognitive Task × Session	7	0.001	84.1	0.500
Box × Session	7	0.007	4.7	<0.001
Box × Cognitive Task	1	<0.001	0.6	0.433

**Table 2:** Significant (*p*<0.05) Pairwise Effect Sizes in Box Displacement between Sessions.

Session	2	3	4	5	6	7	8
1	0.73†	0.86†	1.05†	1.16†	1.27†	1.30†	0.70†
2	-		0.29†	0.39†	0.48†	0.50†	
3	-	-		0.35†	0.35†	0.37†	
4	-	-	-		0.18	0.20	−0.32†
5	-	-	-	-			−0.42†
6	-	-	-	-	-		−0.51†
7	-	-	-	-	-	-	−0.53†
8	-	-	-	-	-	-	-

† *p_adj_*<0.001.

Results of the ANOVA on box velocity indicated statistically significant (*p*<0.05) effects of box, cognitive task, session, and the interaction box × session (see Table 3). Post hoc testing showed that participants could move box 1 with significantly (t = −33.63, DF = 1599, *p_adj_*<0.001, *d* = −0.96) increased velocities than box 2 with a mean velocity of 0.66 cm/s (SE = 0.011 cm/s) versus a mean of 0.30 cm/s (SE = 0.006 cm/s). Presence of the simultaneous cognitive task significantly decreased mean velocity (t = −3.50, DF = 1599, *p_adj_*<0.001, *d* = −0.09) from 0.50 cm/s (SE = 0.010 cm/s) to 0.458 cm/s (SE = 0.010 cm/s). Pairwise comparisons between the 8 sessions of experimentation (see Table 4) indicated several significant differences showing a general increase in box velocity as a function of session until reaching steady state at session 6. Session 1 had significantly decreased velocity relative to all other sessions. Session 7 showed increased velocity relative to sessions 1–5. Session 8 showed lowered velocity relative to sessions 5, 6, and 7, but increased velocity relative to sessions 1, 2, and 3. Trials with both box 1 and box 2 showed increases in velocity as a function of session, but greater increases were seen for box 1 than for box 2.

**Table 3:** ANOVA for Box Velocity.

Factor	DF	η_p_^2^	F	*p*
Session	7	0.152	112.2	<0.001
Cognitive Task	1	0.002	12.2	<0.001
Box	1	0.217	1131.1	<0.001
Cognitive Task × Session	7	0.001	0.8	0.601
Box × Session	7	0.019	14.2	<0.001
Box × Cognitive Task	1	<0.001	0.0	0.914

**Table 4:** Significant (*p*<0.05) Pairwise Effect Sizes in Box Velocity between Sessions.

Session	2	3	4	5	6	7	8
1	0.59†	0.69†	0.91†	1.03†	1.13†	1.28†	0.63†
2	-		0.40†	0.61†	0.74†	0.86†	0.24†
3	-	-	0.32†	0.55†	0.69†	0.80†	0.18
4	-	-	-	0.27†	0.42†	0.50†	
5	-	-	-	-	0.15	0.21†	−0.28†
6	-	-	-	-	-		−0.41†
7	-	-	-	-	-	-	−0.47†
8	-	-	-	-	-	-	-

† *p_adj_*<0.001.

## Discussion

### Effects of the Training on Performance

A primary influence on box displacement and velocity was session, with increases in training time leading to improved performance. Due to the smaller stiffness and thus larger allowable finger displacement in the normal direction of box 1, participants were able to move it further and faster than box 2; however, both boxes showed generally similar trends as a function of session. While interacting with box 1, box displacement showed a large initial jump between sessions 1 and 2 and then showed more gradual increases whereas box velocity showed a more linear trend of improvement. While interacting with box 2, box displacement and velocity performance showed roughly linear gains over the first five sessions before reaching a steady-state These results are generally in agreement with our initial hypothesis that training with vibrotactile feedback would result in increases in performance until reaching steady-state performance, although in the case of box 1 (an easier task) our results indicate that subjects may not have reached steady-state performance in box velocity by session 7.

As hypothesized, when vibrotactile feedback was removed, both box displacement and velocity were decreased relative to steady-state values. However, they did not drop to values as low as those seen during session 1 for either box. Thus, the increases in performance were the result of both increased skill completing the task as well as an increase in the ability to perceive and utilize the vibrotactile feedback. Figure 2 shows the data as a function of session and also plots data from a previous study in which healthy participants interacted with this system in a single session using visual feedback alone [22]. Values of both box displacement and box velocity in these un-trained subjects are smaller than those seen for subjects in the current study using visual feedback alone after seven sessions of training using vibrotactile feedback. Although these data come from a different corpus of subjects, these findings imply that repeated training with vibrotactile feedback may improve motor performance in the absence of this augmentative feedback. However, in the current study a single type of task was used to both train and test performance. In our future work, we will develop a battery of tasks for use so that performance testing can be performed in different tasks than those used for training.

### Effects of the Cognitive Task

Prosthetic hand users have specifically requested haptic feedback as well as relief from visual attention to perform functions [2], [3]. Thus, in the current experiment, motor performance was examined both alone and with a simultaneous cognitive task. Based on our previous work in single-visit experiments [25], we hypothesized that performance on the motor task would be decreased during a simultaneous cognitive task. Although both outcome variables showed a reduction during the performance of a cognitive task, effects were small in comparison to other factors (see Figure 2). Thus, individuals were able to utilize the vibrotactile feedback to perform the task, even in the face of a concurrent cognitive task. This has far-reaching implications for practical application of vibrotactile feedback in users of prosthetic hands who must rely on motor control even when faced with cognitive distraction, such as a simultaneous conversation.

### Impact on Prosthetic Rehabilitation

Using vibrotactile stimulation for prosthetic feedback has obvious pragmatic benefits: it is inexpensive (<$200 for the voice coil used in this study, $1–$5 for more typical vibration motors that could deliver similar stimulation), easy to implement, and non-invasive. However, it has not been widely implemented, possibly due to the lackluster results of previous studies. However, previous studies did not look at ability over periods longer than a single session. The lack of clear benefit from vibrotactile feedback in previous studies could have been a result of insufficient training time. Here we show that users are able to increase motor performance by 3–4 times with just a few days of training. Our results suggest that clinical training protocols for incorporating simple vibrotactile feedback could increase sensorimotor integration and thus could potentially promote wide-scale adoption by users of prosthetic hands. In light of our results, future work to quantify and compare the benefit of augmentative sensory feedback for object manipulation should incorporate multi-day training.

There are a few potential obstacles to adoption of vibrotactile stimulation for sensory feedback, including habituation to the stimulation and audibility of stimulation. Adaptation of sensory afferents to vibrotactile stimuli can be both centrally-mediated and a result of sensory peripheral adaptation. This adaption occurs during continuous vibrotactile stimulation with time constants ranging from 10–40 s, with recovery time constants ranging from 10–30 s [32]. The current study does not show evidence of desensitization, with a monotonic increase of box displacement and velocity during use of the vibrotactile feedback. However, our future work in amputees will study the long-term effects and usability of vibrotactile feedback with prosthetic limb control. The stimulation in this study was provided at 250 Hz, which is in the range of human hearing. When participants produced higher virtual forces, the corresponding increases in amplitude of vibration resulted in an audible sound. Future work will examine potential solutions to this issue. For instance, can this level of noise be reduced through flexible shielding? If not, alternative stimulation frequencies should be explored. Although human glabrous skin is maximally sensitive to 250 Hz stimulation [29], [30], it is also sensitive at lower frequencies at which human hearing thresholds are elevated. A compromise of using a stimulation frequency at 50–80 Hz might mitigate the deleterious auditory effects for listeners without greatly reducing the performance of the user.

The current study has endeavored to answer questions about the role of learning and training time in integrating vibrotactile feedback to perform a motor task with the intention of providing guidance for sensory feedback for prosthetic hand control. However, it must be acknowledged that the participants in this study were intact individuals with working kinesthetic sensation who were asked to perform a three-dimensional task in response to a two-dimensional feedback representation. Thus, although the use of a virtual environment allowed removal of cutaneous cues, kinesthetic cues about finger position were still available to participants and participants could have found the translation of information from three-dimensions to two-dimensions cognitively challenging. For the chosen task, fingertip force was the most relevant cue; however, we cannot remove the possibility that our results seen here in intact individuals do not extend to amputees. However, our recent work has shown that this type of vibrotactile feedback can aid virtual object manipulation performance in individuals using electromyography of the upper limb to interact with the virtual environment [33], and our future work will extend this technique to users of upper-limb prostheses using this paradigm as well as a more realistic paradigm with three-dimensional feedback.

### Summary

Experiments of virtual object manipulation with vibrotactile feedback related to force were conducted across eight sessions over a two-week period. Participants were able to learn to use the vibrotactile feedback to statistically significantly improve object manipulation with training as measured by two performance outcome measures: average box displacement and average velocity. Removal of vibrotactile feedback in session 8 resulted in a reduction in task performance. These results suggest that vibrotactile feedback paired with training may enhance the manipulation ability of prosthetic hand users without the need for more invasive strategies. Our future work will extend to users of upper-limb prostheses and will determine practical methods for implementation of this feedback modality.
